# 15-Deoxy-Δ^12,14^-prostaglandin J_2_ Exerts Antioxidant Effects While Exacerbating Inflammation in Mice Subjected to Ureteral Obstruction

**DOI:** 10.1155/2017/3924912

**Published:** 2017-04-19

**Authors:** Line Nilsson, Fredrik Palm, Rikke Nørregaard

**Affiliations:** ^1^Department of Clinical Medicine, Aarhus University, Aarhus, Denmark; ^2^Division of Integrative Physiology, Department of Medical Cell Biology, Uppsala University, Uppsala, Sweden; ^3^Division of Drug Research, Department of Medical Health Sciences, Linköping University, Linköping, Sweden

## Abstract

Urinary obstruction is associated with inflammation and oxidative stress, leading to renal dysfunction. Previous studies have shown that 15-deoxy-Δ^12,14^-prostaglandin J_2_ (15d-PGJ_2_) has both antioxidant and anti-inflammatory effects. Using a unilateral ureteral obstruction (UUO) mouse model, we examined the effects of 15d-PGJ_2_ on oxidative stress and inflammation in the kidney. Mice were subjected to UUO for 3 days and treated with 15d-PGJ_2_. Protein and RNA expression were examined using immunoblotting and qPCR. 15d-PGJ_2_ increased NF-E2-related nuclear factor erythroid-2 (Nrf2) protein expression in response to UUO, and heme oxygenase 1 (HO-1), a downstream target of Nrf2, was induced by 15d-PGJ_2_. Additionally, 15d-PGJ_2_ prevented protein carbonylation, a UUO-induced oxidative stress marker. Inflammation, measured by nuclear NF-*κ*B, F4/80, and MCP-1, was increased in response to UUO and further increased by 15d-PGJ_2_. Renal injury was aggravated by 15d-PGJ_2_ treatment as measured by kidney injury molecule-1 (KIM-1) and cortical caspase 3 content. No effect of 15d-PGJ_2_ was observed on renal function in mice subjected to UUO. This study illustrates differentiated functioning of 15d-PGJ_2_ on inflammation and oxidative stress in response to obstructive nephropathy. High concentrations of 15d-PGJ_2_ protects against oxidative stress during 3-day UUO in mice; however, it aggravates the associated inflammation.

## 1. Introduction

Obstructive uropathy is a common cause of acute kidney disease and often results from the formation of kidney stones that blocks the flow of urine from the kidney. Ureteral obstruction is characterized by inflammatory cell infiltration and by the accumulation primarily of macrophages in the obstructed kidney, accompanied with increased apoptosis and oxidative stress [1].

Cyclooxygenase 2 (COX-2) is induced in response to unilateral ureteral obstruction (UUO), and COX-2-derived prostaglandins have been associated with the proinflammatory response, primarily prostaglandin E_2_ (PGE_2_) and PGI_2_, which are increased in the early phase of inflammation [2]. 15-Deoxy-Δ^12,14^-prostaglandin J_2_ (15d-PGJ_2_) is derived from prostaglandin D_2_ by dehydration. Increased levels of PGD_2_ and 15d-PGJ_2_ have been identified in the later phase of inflammation; these have been associated with the resolution of inflammation [3, 4]. Increased prostaglandin biosynthesis during both the onset and resolution of inflammation points to a dual role of COX-2 in the inflammatory process. In addition, the administration of a selective COX-2 inhibitor in the late phase of carrageenan-induced pleurisy reduced PGD_2_ and 15d-PGJ_2_ concentrations and exacerbated the inflammatory response, supporting an anti-inflammatory role of COX-2 [3].

15d-PGJ_2_ is also known as a peroxisome proliferator-activated receptor gamma (PPAR*γ*) agonist, and the 15d-PGJ_2_/PPAR*γ* pathway has generally been associated with anti-inflammatory actions [5]. In a number of studies, 15d-PGJ_2_ has been shown to decrease proinflammatory cytokines and reduce organ injury via PPAR*γ* activation [6–8]. In contrast, it has been shown that under physiological concentrations, 15d-PGJ_2_ induced eosinophil migration via PPAR*γ*, suggesting that PPAR*γ* might have biphasic regulatory effects on the immune response [9]. 15d-PGJ_2_ has further been recognized as a potent agonist of the PGD_2_ receptor chemoattractant receptor-homologous molecule expressed on Th2 cells (CRTH2). The CRTH2 receptor has been associated with increased oxidative stress and proinflammatory actions especially in relation to allergic inflammation [10].

Furthermore, 15d-PGJ_2_ itself is chemically reactive and might also form adducts with protein thiol groups and thereby modulate the function of proteins directly [11]. Through this mechanism, 15d-PGJ_2_ has been shown to inhibit activation of the nuclear factor kappa-light-chain-enhancer of activated B cells (NF-*κ*B) transcription pathway, which is a key regulator of proinflammatory mediators. In addition, 15d-PGJ_2_ has been implicated in the activation of the nuclear factor erythroid 2-related factor 2- (Nrf2-) Kelch-like ECH-associated protein 1 (Keap1) signaling pathway, leading to the activation of gene transcription of antioxidant enzymes including heme oxygenase 1 (HO-1) [12].

Given the reports of the dual roles of 15d-PGJ_2_ in regulating both inflammation and oxidative stress, we tested the hypothesis that 15d-PGJ_2_ might protect against UUO-induced damage by modifying inflammation and oxidative stress.

## 2. Materials and Methods

### 2.1. Experimental Animals

All procedures conformed to the Danish National Guidelines for the care and use of experimental animals and to the published guidelines from the USA National Institutes of Health. The animal protocols were approved by the board of the Institute of Clinical Medicine, Aarhus University, corresponding to the licenses for the use of experimental animals issued by the Danish Ministry of Justice.

Studies were performed on adult (10 weeks of age) C57BL/6J mice. Animals had free access to a standard rodent diet (Altromin, Lage, Germany) and tap water. During the experiments, mice were housed in groups of two to three per cage, with a 12 : 12 h light-dark cycle, a temperature of 21 ± 2°C, and a humidity of 55 ± 2%. Animals acclimatized to the cages for 3-4 days before surgery.

### 2.2. Experimental Design and Surgical Procedures

15d-PGJ_2_ was dissolved in dimethyl sulfoxide (DMSO) and diluted with saline to a 4% DMSO concentration. 15d-PGJ_2_ was administered 2 times daily by s.c. injection at low (0.5 mg/kg/day) and high (1 mg/kg/day) doses (*n* = 6). Control groups received vehicle (4% DMSO in saline) (*n* = 6). Treatment was initiated two days prior to surgery and continued throughout the study. At day 3, mice were anesthetized with sevoflurane (3.5% sevoflurane in O_2_/N_2_O mixture, 1.5 l/min) and placed on a heating pad to maintain an appropriate body temperature during surgery. Through a midline abdominal incision, the left ureter was exposed and occluded with a 6-0 silk ligature. UUO was induced for 3 days, where after the mice were sacrificed and the kidneys removed and prepared for quantitative PCR (qPCR) or semiquantitative immunoblotting. Age- and time-matched, sham-operated controls were prepared and observed in parallel with each experimental group (*n* = 5).

### 2.3. Blood Sampling

Three days after UUO, mice were anesthetized with sevoflurane. At the time of sacrifice, a blood sample was taken from the left ventricle, and plasma analysis of creatinine and urea levels was performed using a Roche Cobas 6000 analyzer (Roche Diagnostics).

### 2.4. RNA Isolation and qPCR

Total RNA was purified from the kidney cortex with a NucleoSpin RNA II mini kit following the manufacturer's protocol (Macherey-Nagel, Düren, Germany). RNA was quantitated by spectrophotometry and stored at −80°C. cDNA was synthesized from 0.5 *μ*g purified RNA using the AffinityScript qPCR cDNA synthesis kit (Life Technologies). For qPCR, 100 ng cDNA served as the template for PCR amplification using the SYBR® Green qPCR Master Mix according to the manufacturer's protocol (Life Technologies). mRNA levels were validated by an Aria Mx3000P qPCR System (Agilent Technologies, Santa Clara, CA, USA) with *Gapdh* as a control gene. Primers used for qPCR amplification are specified in Table 1.

### 2.5. Immunoblotting

Nuclear and cytoplasmic extracts were harvested from renal cortical tissue using a Nuclear Extract Kit (Active Motif, Carlsbad, CA, USA) according to manufacturer's protocol. The extracts were used for protein concentration determination and immunoblot analysis, as previously described [13]. Protein concentrations of the extracts were determined using a Pierce BCA protein assay kit (Roche). Briefly, equal amounts of proteins were separated on a 12% Criterion TGX Precast Gel (Bio-Rad Laboratories, København, Denmark) followed by transfer to a Hybond ECL nitrocellulose membrane (GE Healthcare, Hatfield, UK). The membrane was blocked in nonfat dry milk and incubated with primary antibodies (described below) overnight at 4°C. Afterwards, the membrane was incubated with a HRP-conjugated secondary antibody (P448, goat anti-rabbit immunoglobulin, Dako A/S, Glostrup, Denmark). Bound antibody was detected using an enhanced chemiluminescence system (Amersham ECL Plus, GE Healthcare) and visualized on the ChemiDoc MP Imaging System (Bio-Rad). All western blots were normalized to total protein, as measured using Stain-Free technology [14]. GAPDH was examined in parallel and shown as an additional control of equal loading.

### 2.6. Primary Antibodies

Primary antibodies were obtained from the following sources: caspase-3 (Cell Signaling Technology, Danvers, MA, USA, product number 9665, 1 : 500), GAPDH (Cell Signaling, product number 2118, 1 : 2000), HSP90 (Stressgen, San Diego, CA, USA, product number AC88, 1 : 500), histone H3 (Cell Signaling, product number 4499, 1 : 1000), Lamin B (Santa Cruz, product number sc-6217, 1 : 500), HO-1 (Enzo, Farmingdale, NY, USA, product number ADI-SPA-896, 1 : 500), kidney injury molecule-1 (KIM-1) (R&D Systems, Minneapolis, MN, USA, product number AF1817, 1 : 500), NF-*κ*B (Cell Signaling, product number 8242, 1 : 1000), I*κ*B*α* (Cell Signaling, product number 9242, 1 : 1000), Nrf2 (Santa Cruz, product number sc-722, 1 : 500), and Keap1 (Santa Cruz, product number sc-33569, 1 : 500).

### 2.7. Statistics

All data are presented as the means ± SE. Multiple comparisons between experimental groups were performed using a one-way ANOVA followed by post hoc analysis using Bonferroni's multiple comparisons test. GraphPad Prism software (GraphPad Software, La Jolla, CA, USA) was used for all statistical analysis. *P*values < 0.05 were considered significant.

## 3. Results

### 3.1. 15d-PGJ_2_ Promotes Nrf2 Activation during UUO

**Figure 1:**
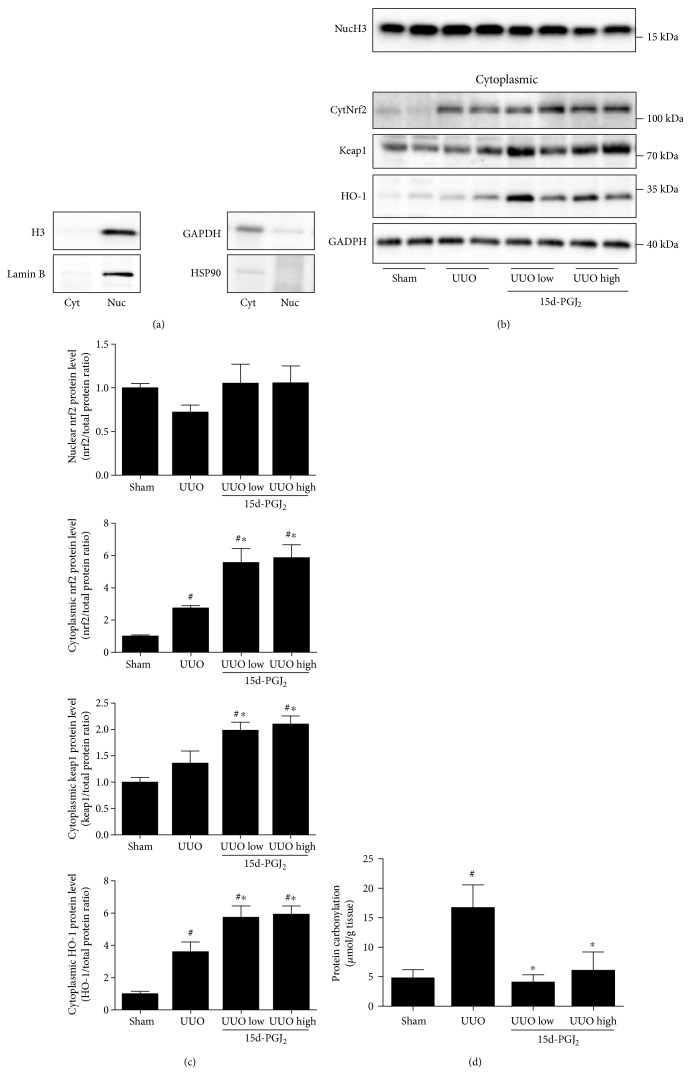
Effects of 15d-PGJ_2_ on the regulation of Nrf2 localization and antioxidants in the kidney cortex during 3-day UUO. (a) Verification of the purity of nuclear and cytosolic extracts. Nuclear markers, histone H3 and Lamin B, as well as cytosolic markers, HSP90 and GAPDH, were used to show the purity of the tissue extracts. (b-c) Nuclear protein extracts were used to analyze Nrf2 nuclear (Nuc) expression. Cytosolic (Cyt) Nrf2, Keap1, and HO-1 proteins were further analyzed in the cytosolic fraction. Representative western blots are shown. (d) Protein carbonylation. Proteins were extracted from cortical tissue and analyzed using 2,4-dinitrophenylhydrazine to quantitate carbonyl content as a marker of protein oxidation. 15d-PGJ_2_ was administered at a low (0.5 mg/kg/day) and a high (1 mg/kg/day) dose as depicted in each graph. Data represents mean ± SEM. ^#^*P* < 0.05 versus that in sham; ^∗^*P* < 0.05 versus that in UUO, using ANOVA and Bonferroni's post hoc test (*n* = 5, sham; *n* = 6, UUO groups). Histone H3 (Hist. H3) and GAPDH are shown as loading controls for nuclear and cytoplasmic protein extracts, respectively. UUO, unilateral ureteral obstruction.

To investigate the cellular localization of proteins of interest, nuclear and cytoplasmic extracts were purified from kidney cortical tissue. In order to detect any contamination of cytoplasmic protein in nuclear fractions and vice versa, we checked the different fractions by western blotting. We used four different antibodies, the anti-H3 and anti-Lamin B to monitor proteins in the nuclear fraction and anti-GAPDH and anti-HSP90 to monitor proteins in the cytoplasmic fraction. As shown in Figure 1(a), we obtained nuclear fractions free of cytoplasmic proteins and vice versa. Our data demonstrated that UUO tended to decrease nuclear Nrf2 and increase cytosolic Nrf2 and Keap1 protein expression. Additional 15d-PGJ_2_ treatment increased both nuclear and cytosolic Nrf2 as well as Keap1 expression, although this was only significant in the cytoplasm (Figures 1(b) and 1(c)). We further investigated the regulation of the Nrf2 target protein HO-1. HO-1 protein expression was increased in response to UUO and further increased in response to 15d-PGJ_2_ treatment (Figures 1(b) and 1(c)). Oxidative stress, estimated from the levels of protein carbonyl groups [15], was increased in response to UUO, and this increase was prevented by 15d-PGJ_2_ administration (Figure 1(d)).

### 3.2. 15d-PGJ_2_ Promotes NF-*κ*B Nuclear Translocation during UUO

**Figure 2:**
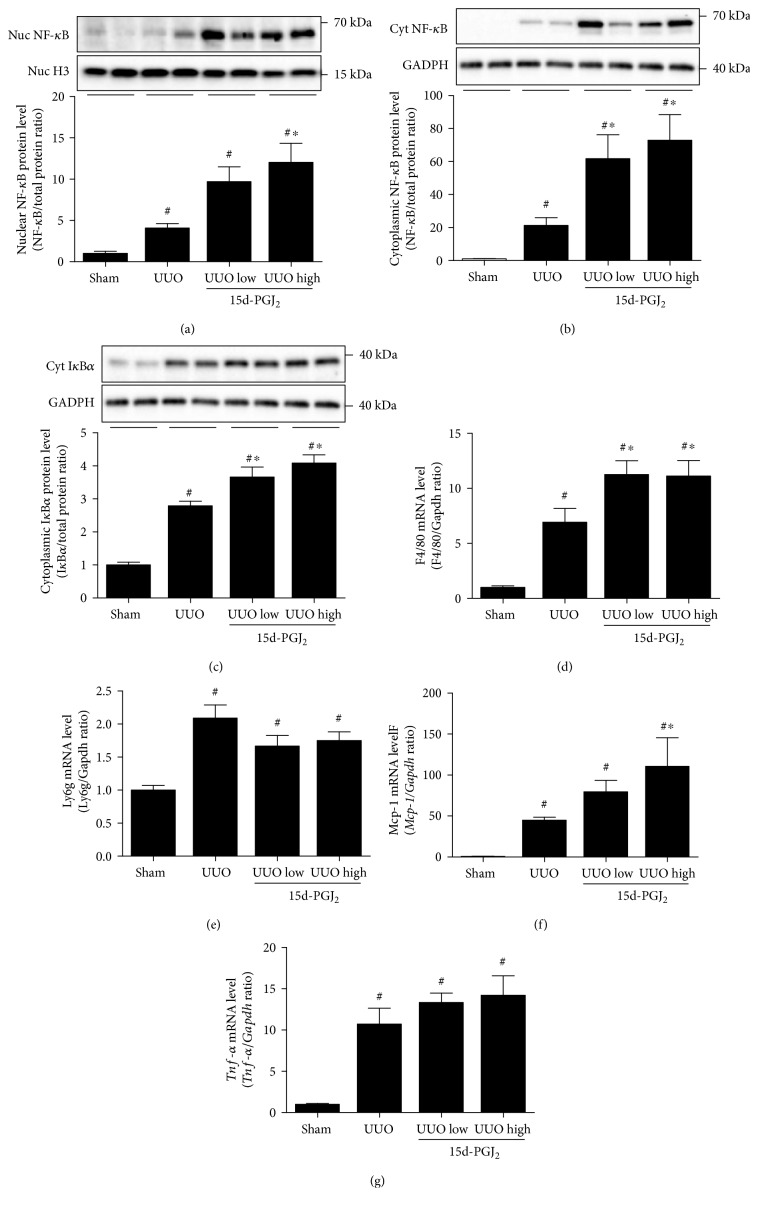
15d-PGJ_2_ enhances the proinflammatory response during 3-day UUO (3dUUO). (a) Nuclear protein extracts were used to analyze cortical NF-*κ*B P65 subunit nuclear protein expression (Nuc). (b) Cortical cytoplasmic protein expression (Cyt) of the NF-*κ*B P65 subunit. (c) Cortical cytoplasmic protein expression of I*κ*B*α*. (d) F4/80 mRNA levels in the kidney cortex. (e) *Ly6G* mRNA levels in the cortex. (f) *Mcp-1* mRNA levels in the cortex. (g) *Tnf-α* mRNA levels in the cortex. Representative blots are shown above each graph. 15d-PGJ_2_ was administered at a low (0.5 mg/kg/day) and a high (1 mg/kg/day) dose as depicted in each graph. Data represents mean ± SEM. ^#^*P* < 0.05 versus that in sham; ^∗^*P* < 0.05 versus that in UUO, using ANOVA and Bonferroni's post hoc tests (*n* = 5, sham; *n* = 6, UUO groups). UUO, unilateral ureteral obstruction.

The activation of NF-*κ*B was investigated based on cellular localization of the NF-*κ*B P65 subunit and on the degradation of the NF-*κ*B inhibitor I*κ*B*α* which plays a role for the nuclear translocation and activation of NF-*κ*B [16]. NF-*κ*B was induced in both nuclear and cytoplasmic fractions in response to UUO and further increased after 15d-PGJ_2_ administration, indicating a proinflammatory effect of 15d-PGJ_2_ (Figures 2(a) and 2(b)). However, western blotting of cytoplasmic I*κ*B*α* demonstrated that NF-*κ*B activation induced by both UUO and treatment of 15d-PGJ_2_ was not associated with I*κ*B*α* degradation (Figure 2(c)). To further evaluate the inflammatory response, immune cell infiltration and inflammatory markers were analyzed. mRNA levels of the macrophage marker F4/80 were increased in response to UUO and were further increased in response to 15d-PGJ_2_ (Figure 2(d)). mRNA levels of neutrophil markers *Ly6G* and *Mcp-1* were likewise increased in response to UUO, where 15d-PGJ_2_ further increased the *Mcp-1* mRNA expression at a high dose (Figures 2(e) and 2(f)). *Tnf-α* mRNA levels were increased in response to UUO and slightly induced by 15d-PGJ_2_ treatment (Figure 2(g)).

### 3.3. 15d-PGJ_2_ Worsens the Renal Injury Following UUO

**Figure 3:**
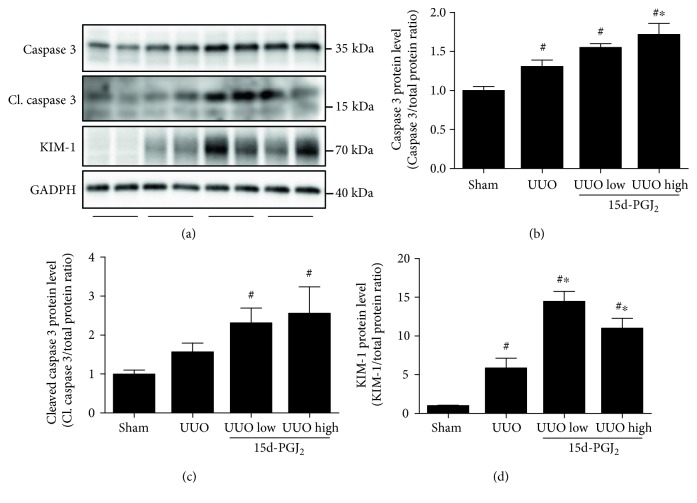
Cortical apoptosis and kidney injury are aggravated during UUO by treatment with 15d-PGJ_2_. (a) Caspase 3 protein expression. (b) Cleaved caspase 3 (Cl. caspase 3) protein expression. (c) KIM-1 protein expression. Representative blots are shown above each graph. 15d-PGJ_2_ was administered at a low (0.5 mg/kg/day) and a high (1 mg/kg/day) dose as depicted in each graph. Data represents mean ± SEM. ^#^*P* < 0.05 versus that in sham; ^∗^*P* < 0.05 versus that in UUO, using ANOVA and Bonferroni's post hoc tests (*n* = 5, sham; *n* = 6, UUO groups).

15d-PGJ_2_ can exert proapoptotic functions through the activation of several proapoptotic mediators including caspase 3 [17, 18]. Caspase 3 was increased in response to UUO and further increased by 15d-PGJ_2_ treatment (Figure 3(a)). Furthermore, cleaved caspase 3 protein abundance increased significantly in response to 15d-PGJ_2_ (Figure 3(b)). The response of KIM-1, a general injury marker which regulates inflammation [19], to UUO was also analyzed in the cortex, and KIM-1 protein expression was found to be upregulated after UUO and further increased by 15d-PGJ_2_ treatment (Figure 3(c)).

### 3.4. 15d-PGJ_2_ Receptors Are Upregulated in Response to UUO

**Figure 4:**
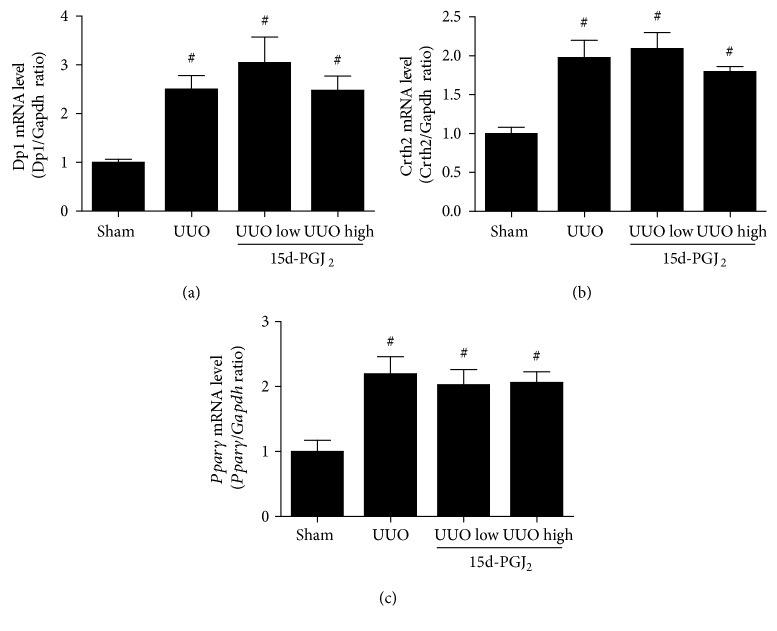
15d-PGJ_2_ receptor expression is increased in response to UUO. mRNA was purified from kidney cortical tissue and analyzed using qPCR. (a) *Dp1* mRNA levels in response to UUO. (b) *Crth2* mRNA levels in response to UUO. (c) *Pparγ* mRNA levels in response to UUO. 15d-PGJ_2_ was administered at a low (0.5 mg/kg/day) and a high (1 mg/kg/day) dose as depicted in each graph. Data represents mean ± SEM. ^#^*P* < 0.05 versus that in sham, using ANOVA and Bonferroni's post hoc tests (*n* = 5, sham; *n* = 6, 3-day UUO groups). UUO, unilateral ureteral obstruction.

In order to investigate the receptors responsible for the effect of 15d-PGJ_2_ during UUO, we investigated the expression of known 15d-PGJ_2_ receptors in response to UUO. The mRNA levels of the receptors *Dp1*, *Crth2*, and *Pparγ* were upregulated in response to UUO, whereas no change in receptor mRNA expression was found in response to 15d-PGJ_2_ treatment (Figures 4(a), 4(b), and 4(c)).

### 3.5. 15d-PGJ_2_ Has No Effect on Renal Function in Response to UUO

The effect of 15d-PGJ_2_ on renal function following UUO was evaluated by analyzing kidney weight, plasma creatinine, and BUN (Table 2). 15d-PGJ_2_ treatment had no effect on kidney weight and plasma creatinine in UUO mice, but a slight increase in BUN was observed with a high dose of 15d-PGJ_2_.

## 4. Discussion

The main findings of the present study were that high concentrations of 15d-PGJ_2_ attenuated oxidative stress and that this was associated with increased HO-1 abundance in the obstructed kidney, which might be mediated through increased Nrf2 nuclear translocation. However, the UUO-induced inflammatory response as well as apoptosis was aggravated by high concentrations of 15d-PGJ_2_ as demonstrated by increased levels of proinflammatory markers as well as KIM-1 and activation of the caspase 3.

### 4.1. 15d-PGJ_2_ Induces Nrf2 Nuclear Translocation

Oxidative stress is induced in response to UUO, mainly owing to the suppression of antioxidant enzymes [20]. Nrf2 is a transcription factor that regulates cytoprotective proteins including antioxidants such as HO-1 and plays a major role in protection against oxidative damage [21]. Consistent with previous studies [22], we showed that UUO decreases nuclear Nrf2 and increases cytosolic Nrf2 expression resulting in increased oxidative stress. This study supports the role of 15d-PGJ_2_ in activation of the Nrf2 pathway and in antioxidant defense against UUO-induced oxidative stress. 15d-PGJ_2_ has been shown to activate Nrf2 by covalent modification of the thiol groups of endogenous Keap1, releasing Nrf2 from the Keap1-Nrf2 complex [11]. Thereupon, Nrf2 freely translocates into the nucleus, leading to the transcription of antioxidant genes. However, 15d-PGJ_2_ treatment did also increase the renal expression of Keap1 and Nrf2 in the cytoplasm. The reason for this discrepancy remains unclear, but it has previously been suggested that cytosolic Keap1 and Nrf2 protein levels may vary according to experimental models of oxidative stress and the severity of renal injury [23, 24]. One could therefore speculate that other effects of 15d-PGJ_2_ or perhaps other pathways can be dominating in this UUO model and play a role in facilitating nuclear translocation rather than directly inducing dissociation of the Nrf2/Keap1 complex. Several in vitro studies have suggested that the protective effects of 15d-PGJ_2_ on renal tubular cells might be mediated via the Nrf2 pathway [25–27]. In addition, it has been shown that the 15d-PGJ_2_-induced HO-1 levels in serum were absent in Nrf2 KO mice subjected to hepatic ischemia, indicating that the Nrf2 pathway might mediate HO-1 induction in response to 15d-PGJ_2_ treatment [28]. However, HO-1 expression was also induced in nontreated UUO mice, so we cannot exclude the possibility that other factors might be involved in the induction of HO-1. The previous studies have demonstrated that HO-1 expression can be induced by other stimuli such as cytokines, NO, and growth factors, and several of these factors are increased in response to renal injury [29]. In addition, our data showed that increased protein carbonylation, a marker of oxidative stress, in response to UUO was prevented by 15d-PGJ_2_, further supporting that 15d-PGJ_2_ has antioxidant effects on mice subjected to UUO.

### 4.2. Enhanced NF-*κ*B Nuclear Translocation in Response to 15d-PGJ_2_

Inflammation also plays a major role in the progression of obstructive nephropathy, and the NF-*κ*B pathway is known to be activated during UUO [30]. 15d-PGJ_2_ has been described as an anti-inflammatory prostaglandin in part owing to its ability to form adduct with the I*κ*B kinase (IKK) to prevent phosphorylation and degradation of I*κ*B*α* which binds to the NF-*κ*B p65 subunit and inhibits NF-*κ*B nuclear translocation [31], and the activation of NF-*κ*B in response to renal I/R was shown to be attenuated by 15d-PGJ_2_ administration in rats [32]. In the present study, we observe increased cytosolic NF-*κ*B protein levels and reduced degradation of I*κ*B*α* after 15d-PGJ_2_ administration suggesting that 15d-PGJ_2_ prevents translocation of NF-*κ*B from the cytoplasm indicating that 15d-PGJ_2_ might have anti-inflammatory properties. In contrast, our data showed that 15d-PGJ_2_ treatment can also induce nuclear NF-*κ*B expression in response to UUO, which argues against an anti-inflammatory function. Although I*κ*B*α* is not degraded, I*κ*B*α* is most likely modified in some way, which can cause I*κ*B*α* to dissociate from NF-*κ*B, exposing a nuclear localization signal as a prerequisite to nuclear translocation [33]. This result was further supported by the observation of increased macrophage infiltration and *Mcp-1* expression in the kidneys of UUO mice. In addition, it had previously been suggested that KIM-1 can increase *Mcp-1* expression and regulate the immune response of inflammatory cells [19]. So we cannot rule out the possibility that the 15d-PGJ_2_-induced KIM-1 expression in UUO mice might also play a role in increasing the expression of *Mcp-1* and infiltration of macrophages.

In vitro studies on murine macrophages have shown that 15d-PGJ_2_ retains NF-*κ*B in the cytoplasm during LPS stimulation [34]. Furthermore, previous studies have demonstrated that 15d-PGJ_2_ might induce NF-*κ*B activity at low levels but directly inhibit NF-*κ*B at higher levels [35]. Therefore, the pro- and anti-inflammatory functions of 15d-PGJ_2_ might be concentration-dependent. This phenomenon could be important for the proinflammatory function of 15d-PGJ_2_ we observed during UUO. For example, 15d-PGJ_2_ has been shown to induce oxidative stress in LPS-stimulated macrophages, which was associated with increased apoptosis and which might represent a mechanism underlying the 15d-PGJ_2_-derived resolution of inflammation [36]. However, most studies investigating apoptosis in response to 15d-PGJ_2_ were required to use very high concentrations of 15d-PGJ_2_ (*μ*M range) in order to affect cell viability in vitro [17, 37].

This study is based on an in vivo model, which is a complex model of obstructive kidney disease. The previous studies showing pro- and anti-inflammatory functions of 15d-PGJ_2_ are predominantly in vitro studies, which are not directly comparable to our model. Increased inflammation in response to 15d-PGJ_2_ treatment may be concentration-dependent. However, we cannot rule out the possibility that off-target effects might occur due to the high concentration of 15d-PGJ_2_ used within this study. In addition, during inflammation, other proinflammatory prostaglandins as PGE_2_ and TXA_2_ might also be produced in excess after UUO. If the system is oversaturated with proinflammatory mediators, the addition of 15d-PGJ_2_ could only offer little or no help resulting in increased inflammation.

15d-PGJ_2_ has been identified as an agonist for several receptors including PPAR*γ*, DP1, and CRTH2. In this study, we showed increased expression of these 15d-PGJ_2_ receptors in response to UUO. Previously, 15d-PGJ_2_ has been shown to exert anti-inflammatory functions through PPAR*γ*, for example, by the inactivation of NF-*κ*B [38]. In contrast, the activation of CRTH2 plays an important role in allergic inflammation [39]. However, it has recently been demonstrated that administration of a CRTH2 antagonist to mice subjected to UUO suppresses the progression of inflammation and fibrosis [40] indicating that activation of the CRTH2 pathway might likewise play a role in UUO-induced injury. Taking into account that 15d-PGJ_2_ might show proinflammatory effects on the obstructed kidney, the role of CRTH2 in relation to UUO-induced injury should be investigated. However, further studies will be needed to clarify the role of each receptor in the 15d-PGJ_2_-induced regulation of antioxidant defense and inflammation identified in this study.

**Figure 5:**
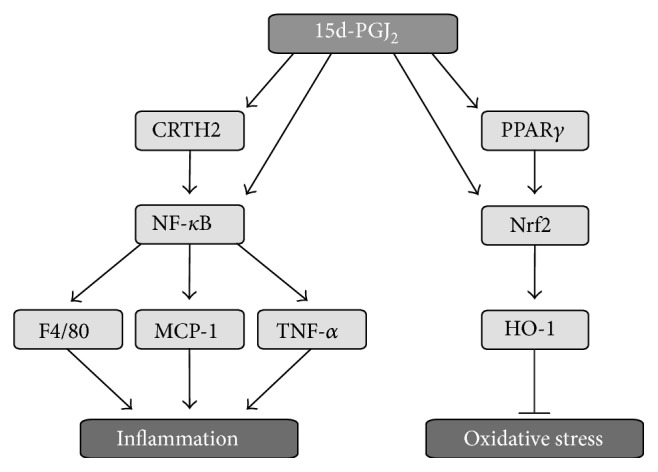
Potential mechanisms involved in the antioxidant and proinflammatory actions of 15d-PGJ_2_. Ureteral obstruction induces oxidative stress and inflammation. We observed that 15d-PGJ_2_ prevents the increase in oxidative stress, probably through activation of the transcription factor Nrf2 and its downstream target HO-1. PPAR*γ* has previously been shown to activate Nrf2 and might therefore be involved in oxidative protection. 15d-PGJ_2_ was shown to exacerbate inflammation through the activation of NF-*κ*B and its downstream targets MCP-1 and TNF-*α* as well as via macrophage infiltration (F4/80). CRTH2 has been shown to activate the inflammatory response and might be involved in the 15d-PGJ_2_-mediated activation of NF-*κ*B.

In summary, this study demonstrated that high concentration of 15d-PGJ_2_ decreased oxidative stress-induced damages in response to UUO through the activation of Nrf2. However, the inflammatory response induced by UUO, estimated from nuclear NF-*κ*B abundance and macrophage infiltration, was worsened by higher concentrations of 15d-PGJ_2_ treatment (Figure 5).

## 5. Conclusion

Our results indicate that high concentrations of 15d-PGJ_2_ may exert both antioxidant and inflammatory actions in mice which have been subjected to UUO for 3 days. Since accumulating evidence points towards a potential therapeutic use of 15d-PGJ_2_, our findings exhibit the need for a detailed understanding of its targets and mechanisms of action in characterized settings in order to confirm its beneficial effects.

## Figures and Tables

**Table 1:** Primers used for qPCR amplification.

Gene/protein (accession number)^∗^	Forward primer (5′–3′)	Reverse primer (5′–3′)
*Mouse*		
CRTH2 (NM_009962.3)	GCGCTATCCGACTTGTTAGC	TGAGGAAGAAGACCGAGGAA
DP1 (NM_008962.4)	CTTGCGTTTCCTGTCTGTGA	ATGGCTGCTCCAGTTTCTTGT
F4/80 (NM_010130.4)	TGCTAGTGGAGGCAGTGATG	CTGTATTCAACCAGCAGCGA
GAPDH (NM_008084.3)	GACGGCCGCATCTTCTTGTG	GCGCCCAATACGGCCAAATC
Ly6G (NM_148939.2)	ACTCCTGGTCCAGTGCTCAG	GGCCCTCAGACACCTTATGA
MCP-1 (NM_011333.3)	CCCAATGAGTAGGCTGGAGA	TCTGGACCCATTCCTTCTTG
PPAR*γ* (NM_011146.3)	CCCTGGCAAAGCATTTGTAT	GAAACTGGCACCCTTGAAAA
TNF-*α* (NM_013693.3)	AGGCTGCCCCGACTACGT	GACTTTCTCCTGGTATGAGATAGCAAA

^∗^GenBank accession numbers.

**Table 2:** Effect of 15d-PGJ2 on body weight, kidney weight, and functional plasma parameters.

	Sham	UUO	UUO + low 15d-PGJ_2_	UUO + high 15d-PGJ_2_
Body weight, g	21.6 ± 0.8	19.3 ± 1.0	20.0 ± 0.5	19.8 ± 0.5
Obstructed KW, mg/20 g BW	113.3 ± 4.7	184.8 ± 3.1^#^	176.7 ± 3.1	180.9 ± 3.2
Plasma creatinine, *μ*mol/l	7.4 ± 0.7	9.3 ± 0.9	10.5 ± 0.9	8.3 ± 0.4
BUN, mmol/l	8.9 ± 0.3	8.6 ± 0.3	9.1 ± 0.2	11.2 ± 0.6^∗^

Biochemical values. Mice were subjected to UUO for 3 days (UUO) and treated with 15d-PGJ2 at a low dose (0.5 mg/kg/day) and a high dose (1 mg/kg/day). At the time of sacrifice, a blood sample was taken from the left ventricle. Body weight and obstructed kidney weight were measured. Plasma creatinine and BUN were measured using Roche Cobas 6000 analyzer (Roche Diagnostics, Hvidovre, Denmark). Values are means ± SEM, *n* = 6. ^#^*P* < 0.05 versus that in sham. ^∗^*P* < 0.05 versus that in UUO. 15d-PGJ2: 15-deoxy-Δ^12,14^-prostaglandin J_2_; BW: body weight; KW: kidney weight; BUN: blood urea nitrogen.
